# Identification of elite fiber quality loci in upland cotton based on the genotyping-by-target-sequencing technology

**DOI:** 10.3389/fpls.2022.1027806

**Published:** 2022-11-03

**Authors:** Hong Chen, Zegang Han, Qi Ma, Chengguang Dong, Xinzhu Ning, Jilian Li, Hai Lin, Shouzhen Xu, Yiqian Li, Yan Hu, Zhanfeng Si, Qingping Song

**Affiliations:** ^1^ Cotton Research Institute, Xinjiang Academy of Agricultural and Reclamation Science, Northwest Inland Region Key Laboratory of Cotton Biology and Genetic Breeding of Ministry of Agriculture, Shihezi, China; ^2^ Zhejiang Provincial Key Laboratory of Crop Genetic Resources, The Advanced Seed Research Institute, Plant Precision Breeding Academy, College of Agriculture and Biotechnology, Zhejiang University, Hangzhou, China

**Keywords:** genotyping-by-target-sequencing (GBTS), upland cotton, fiber quality, association studies, single nucleotide polymorphism (SNP)

## Abstract

Genome-wide association studies (GWAS) of fiber quality traits of upland cotton were conducted to identify the single-nucleotide polymorphic (SNP) loci associated with cotton fiber quality, which lays the foundation for the mining of elite] cotton fiber gene resources and its application in molecular breeding. A total of 612 upland cotton accessions were genotyped using the ZJU Cotton Chip No. 1 40K chip array *via* the liquid-phase probe hybridization-based genotyping-by-target-sequencing (GBTS) technology. In the present study, five fiber quality traits, namely fiber length, fiber strength, micronaire, uniformity and elongation, showed different degrees of variation in different environments. The average coefficient of variation of fiber strength was the greatest, whereas the average coefficient of variation of uniformity was the least. Significant or extremely significant correlations existed among the five fiber quality traits, especially fiber length, strength, uniformity and elongation all being significantly negative correlated with micronaire. Population cluster analysis divided the 612 accessions into four groups: 73 assigned to group I, 226 to group II, 220 to group III and 93 to group IV. Genome-wide association studies of five fiber quality traits in five environments was performed and a total of 42 SNP loci associated with target traits was detected, distributed on 19 chromosomes, with eight loci associated with fiber length, five loci associated with fiber strength, four loci associated with micronaire, twelve loci associated with fiber uniformity and thirteen loci associated with fiber elongation. Of them, seven loci were detected in more than two environments. Nine SNP loci related to fiber length, fiber strength, uniformity and elongation were found on chromosome A07, seven loci related to fiber length, fiber strength, micronaire and elongation were detected on chromosome D01, and five loci associated with fiber length, uniformity and micronaire were detected on chromosome D11. The results from this study could provide more precise molecular markers and genetic resources for cotton breeding for better fiber quality in the future.

## Introduction

Cotton is a significant economic crop in the world and occupies a pivotal position in China’s national economy. The use of modern biotechnology to identify elite genetic resources of cotton is of great significance for the breeding of new superior cotton varieties. In recent years, the completion of the whole-genome sequencing of the D genome of the wild cotton species *Gossypium raimondii*, the A genome of Asian cotton (*Gossypium arboreum*), the whole-genome sequencing and assembly of allotetraploid upland cotton (*Gossypium hirsutum*) and island cotton (*Gossypium barbadense*) (Paterson et al., 2012; Wang and Ruan, 2012; Li et al., 2014; Li et al., 2015; Zhang et al., 2015; Hu et al., 2019; Wang et al., 2019; Ma et al., 2021) laid the foundation for the identification of important trait loci and deep mining of genes in cotton. With the maturity of genome sequencing technology and the reduction of sequencing costs, millions of single-nucleotide polymorphic (SNP) loci have been screened and identified by re-sequencing many cotton accessions (Wang et al., 2015; Hu et al., 2019). Based on efficient and low-cost SNP genotyping technology, identification of loci significantly related to important traits and functional genes in cotton populations *via* genome-wide association studies (GWAS) have become more precise and effective (Fang et al., 2017; Wang et al., 2020; Zhang et al., 2020). For instance, Fang et al. (2017) re-sequenced 318 upland cotton accessions and identified 119 SNP loci through GWAS, 71 of which were related to fiber yield, 45 to fiber quality, and three to resistance to Verticillium wilt (Fang et al., 2017). He et al. (2021) comprehensively identified and evaluated the sources and aggregation effects of fiber quality-related superior allelic variation in the current cultivated upland cotton gene pool by analyzing data from 1,245 upland cotton genotypes and nearly 80,000 fiber quality data. The germplasm carrying the multi-locus combination has significantly improved fiber length and fiber strength. The insufficient number of superior allelic variants may be the key reason for the current bottleneck in the improvement of upland cotton fiber quality (He et al., 2021). Ma et al. (2021) re-sequenced 1,081 upland cotton accessions around the world (average 10.65× depth) and identified 304,630 structural variants using *G. hirsutum* ‘NDM8’ as the reference genome, as well as loci affecting fiber length, strength, boll weight, and lint percentage obtained from large-scale environmental assessment and Verticillium wilt resistance data analysis, and found 446 structural variants significantly associated with important traits. The loci affecting fiber quality traits were mainly located in sub-genome D, whereas the loci affecting yield traits were mainly located in sub-genome A. Among the 907 genes related to fiber quality and yield, 84.23% were expressed in different developmental stages of fiber. An important new gene, *GhNCS*, controlling Verticillium wilt resistance was also discovered (Ma et al., 2021). In addition, Sun et al., 2017 conducted phenotypic identification and GWAS on 719 domestic and foreign upland cotton varieties under multi-site environmental conditions over a number of years using high-throughput SNP array and cutting-edge technologies such as GWAS and transcriptome sequencing, and they detected 46 SNP loci significantly associated with five major traits of cotton fiber quality, including fiber length, strength and micronaire. Among these, 20 SNPs related to fiber length were distributed on chromosomes A07, A10, D03, D05, D06, D07 and D11, seven of which were located on A07 and four of which were located on D11. Eighteen SNPs related to fiber strength were distributed on chromosomes A01, A07, A13, D06, D10, D11 and D13, seven of which were distributed on A07 (Sun et al., 2017). Huang et al. (2017) performed genotyping on 503 upland cotton accessions from China using a cotton 63K array and a corresponding high-density genetic map, and found that the upland cotton group could be clearly divided into three subgroups, with the division of subgroups not being directly related to the geographical origin and breeding period, suggesting that there were cotton introductions and gene exchanges between different main cotton-growing regions in China. A total of 160 associated quantitative trait loci (QTLs) and 158 candidate intervals were detected by GWAS on 16 traits, and one candidate gene (*Gh_D08G2376*) related to cotton lint percentage was found (Huang et al., 2017). Dong et al. (2019) conducted GWAS, using a cotton 80K array, and 23 loci associated with fiber quality were detected and 128 candidate genes were identified (Dong et al., 2019).

In the current study, in order to further investigate elite allelic variation and genetic resources related to fiber quality traits of upland cotton, field experiments comparing 612 upland cotton accessions from different cotton-producing regions were carried out in five environments at two locations in China, Korla in southern Xinjiang and Shihezi in northern Xinjiang over three years to obtain data on fiber quality traits. Genome-wide scanning and association studies were performed using the liquid-phase probe hybridization-based genotyping-by-target-sequencing technology to identify stably SNP loci related to fiber quality traits in different environments. The results should lay a foundation for the mining of elite cotton fiber gene resources and its application in breeding improved cotton varieties.

## Materials and methods

### Test materials and field experiments

A total of 612 upland cotton accessions were used as the test materials, of which 378 were from the Northwest inland cotton-producing region, 125 were from the Yellow River Basin cotton-producing region, 68 were from the Yangtze River Basin cotton-producing region, 19 were from the northern extra-early cotton-producing region and 22 were introduced from abroad. All accessions were planted in Shihezi Experimental Base in northern Xinjiang (85.94°E, 44.27°N) and Korla Experimental Base in southern Xinjiang (86.06°E, 41.68°N) of Xinjiang Academy of Agricultural and Reclamation Science in 2019 and 2020, and in Shihezi Experimental Base in 2021 only. A randomized, complete block design was adopted in the field experiments. The test plots were sown as double-rows with three replicates. Spacing between the double-row areas was 66 cm, the row length was 3 m, and the average within-row plant spacing was 10 cm. Sowing was performed manually and drip irrigation cultivation under film was carried out. Field management was performed using local conventional methods.

### Phenotypic data analysis

Twenty bolls opened naturally from each cultivar were hand-harvested in the cotton bolls open stage. A total of 15 g of lint cotton was collected from each replicate plot and the fiber quality were examined in the Northwest Inland Region Key Laboratory of Cotton Biology and Genetic Breeding of Ministry of Agriculture, using a HFT9000 Fiber Tester (Premier Inc, Coimbatore, India). Traits measured included the fiber length(FL, mm), fiber strength (FS, cN/Tex), fiber micronaire (FM), fiber uniformity (FU, %) and fiber elongation (FE, %).

Excel 2010 and SPSS 19 were used for statistical analysis of phenotypic data. Mean phenotypic values (mean) were calculated and the best linear unbiased predictions (blup) were estimated for each trait in the five environments. Therefore, a total of seven phenotypic data sets, 2019SHZ, 2019KRL, 2020SHZ, 2020KRL, 2021SHZ, mean and blup were statistically analyzed.

### Genome-wide association studies (GWAS)

DNA was extracted from young leaves of each test material by the CTAB method (Paterson et al., 1993; Barrett et al., 2005). The “ZJU Cotton Array No. 1” 40K array, jointly designed and developed by Zhejiang University and Boruidi Bio-Co., Ltd., based on liquid-phase probe hybridization-based genotyping-by-target-sequencing technology (Si et al., 2022), was used to perform genotyping detection. The array consisted of 40,523 core SNP markers, which were evenly distributed on the 26 chromosomes of allotetraploid cotton. After excluding those SNPs with minor allele frequency (MAF) < 0.05, the missing rate ≥ 0.3 and the same as the reference genome of cultivar ‘TM-1’, 30,309 high-quality SNP markers were finally determined for further research. GWAS were performed based on the mixed linear model (MLM) method in the EMMAX package (Kang et al., 2010). The significance threshold of the *P* value was determined as described in previous studies (Cai et al., 2017; Sun et al., 2017; Wang et al., 2017), that is, using the threshold *P* ≤ 1/n, so the threshold (*P*) of 30,309 SNPs for association analysis was 4.48 [*P* ≤ 1/30,309 = 3.29×10^−5^, *P*=−log10(*P*) ≥ 4.48]. Therefore, the threshold of *P*=4.5 was used in this study to screen for GWAS-associated loci. Manhattan plots were created using the R language package ‘qqman’.

## Results

### Phenotypic variation analysis of fiber quality traits

The variation of each of the five cotton fiber quality traits was normally distributed (**Supplementary Figure 1**). The results of phenotypic variation analysis showed that the five traits exhibited varying degrees of variation in different environments. The variation ranges of fiber length, fiber strength, micronaire, fiber uniformity and fiber elongation were 21.70~34.74 mm, 20.98~43.54 cN/Tex, 2.77~5.73, 77.10~89.08%, and 6.20~7.20%, respectively. The average coefficients of variation were 6.83%, 10.75%, 9.42%, 1.55% and 2.08%, respectively. Of these, the average coefficient of variation for fiber strength was the highest, being most affected by the environment, at 10.75%, and that of variation of uniformity was the lowest at 1.55% (**Supplementary Table 1**).

### Analysis of variance and heritability of fiber quality traits

Variance analysis showed that there were extremely significant differences among accessions and environments and in interactions between accessions and environments. The heritability of FL, FS, FM, FU and FE were 84.94%, 87.07%, 95.25%, 45.10% and 50.49% respectively, among which the highest heritability was of micronaire (95.25%), and the lowest heritability was of uniformity (45.10%) (**Supplementary Table 2**).

### Correlation analysis of fiber quality traits

Correlation analysis of fiber length, fiber strength, micronaire, uniformity and elongation were carried out. Fiber length was highly significantly correlated with strength, micronaire, uniformity and elongation, and the correlation coefficients were 0.898, -0.535, 0.562 and 0.815, correspondingly. Fiber strength was extremely significantly correlated with micronaire, uniformity and elongation, and the correlation coefficients were -0.454, 0.642 and 0.795, respectively. Micronaire was significantly negatively correlated with uniformity and elongation, and the correlation coefficients were -0.012 and -0.321, respectively. Fiber uniformity and elongation were extremely significantly positively correlated with one another (**Table 1**).

**Table 1:** Correlation coefficients between fiber quality traits.

	FL	FS	FM	FU	FE
FL	–	0.898^**^	-0.535^**^	0.562^**^	0.815^**^
FS	0.898^**^	–	-0.454^**^	0.642^**^	0.795^**^
FM	-0.535^**^	-0.454^**^	–	-0.102^*^	-0.321^**^
FU	0.562^**^	0.642^**^	-0.102^*^	–	0.660^**^
FE	0.815^**^	0.795^**^	-0.321^**^	0.660^**^	–

* and ** indicate significance at P = 0.05 and P = 0.01 levels, respectively.

### Population structure analysis

**Figure 1:**
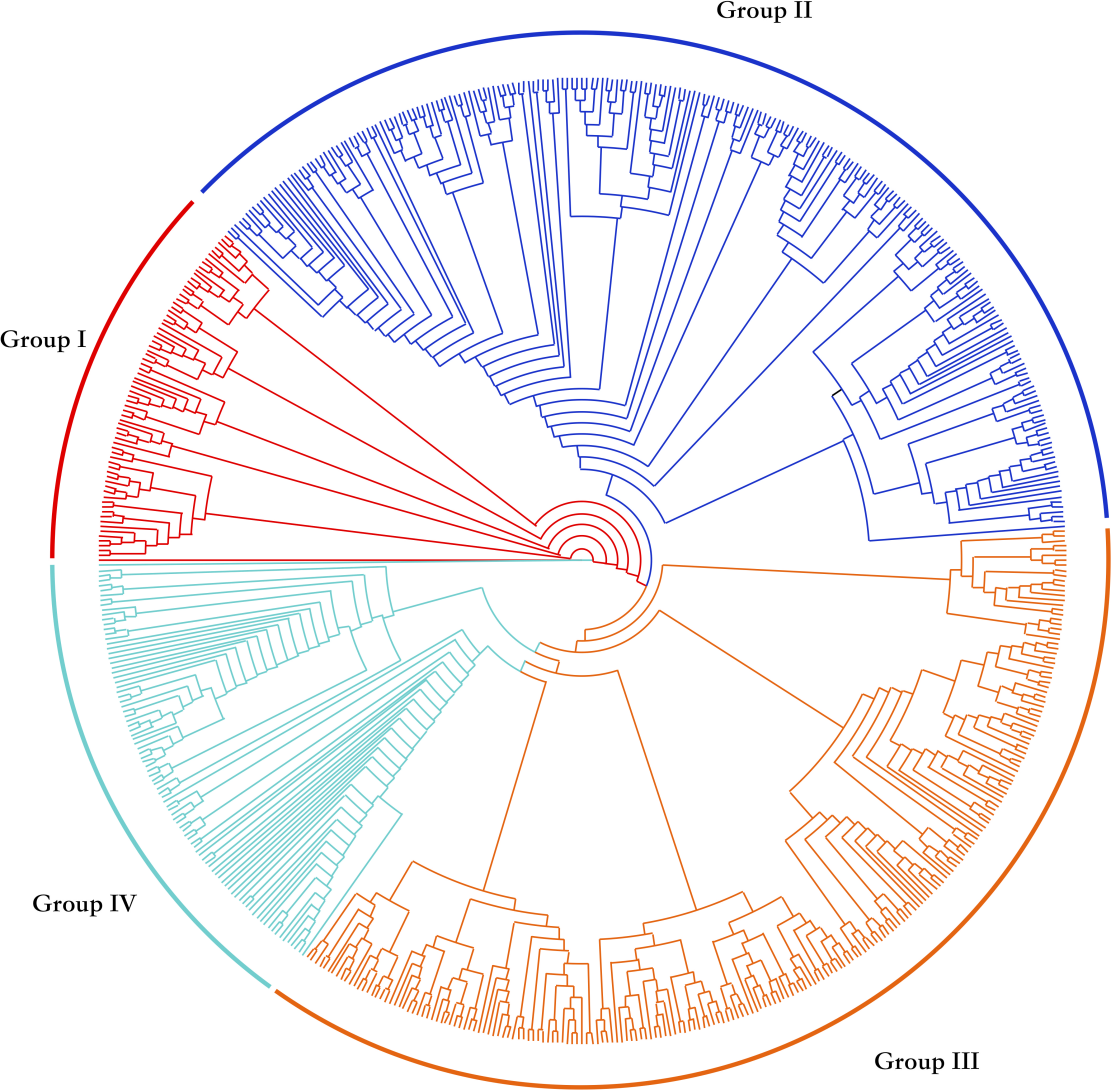
Cluster analysis of population relationship among 612 accessions.

Through cluster analysis of population relationships, the 612 upland cotton accessions were divided into four groups: I, II, III and IV (**Figure 1**). Seventy-three cultivars were classified in group I (red), 226 in group II (blue), 220 in group III (yellow) and 93 in group IV (green) (**Table 2**). Of the accessions from the​​ Yellow River Basin cotton-producing region, 6.4%, 60.0%, 15.2% and 18.4% were distributed in groups I, II, III and IV respectively; with 1.5%, 63.2%, 16.2% and 19.1% of the accessions from the Yangtze River Basin cotton-producing region being distributed in groups I, II, III and IV respectively. Of the accessions from the northern extra-early cotton-producing region, 15.8%, 31.6%, 47.4% and 5.3% were distributed in groups I, II, III and IV, respectively, whereas 0.0%, 59.1%, 18.2% and 22.7% of the accessions introduced from abroad were distributed in groups I, II, III and IV respectively. Moreover, with 16.1%, 23.5%, 46.8% and 13.5% of the accessions from the Northwest inland cotton-producing region being distributed in groups I, II, III and IV, respectively. In summary, approximately 60% of the accessions from the Yellow River Basin, the Yangtze River Basin and from abroad were clustered in group II. Although the accessions from the Northwest inland cotton-producing region were distributed in all four groups, a large number of these accessions were mainly clustered in group III. The genetic relationship between groups showed that the cultivars from the Northwest inland cotton-producing region were distributed in various groups, consistent with the history of cotton breeding in China, where the superior germplasm from other cotton-producing regions in China was widely introduced to enrich the genetic background of cotton in the Northwest inland cotton-producing region, resulted in a closer relationship with the accessions from other cotton-producing regions in China.

**Table 2:** Distribution of accessions from five sources among the four groups.

Group	Total number	Yellow River Basin	Yangtze River Basin	Northern extra-early cotton- producing region	Introduced from abroad	Northwest, inland cotton- producing region
I	73	8	1	3	0	61
II	226	75	43	6	13	89
III	220	19	11	9	4	177
IV	93	23	13	1	5	51

### GWAS of fiber quality traits

**Figure 2:**
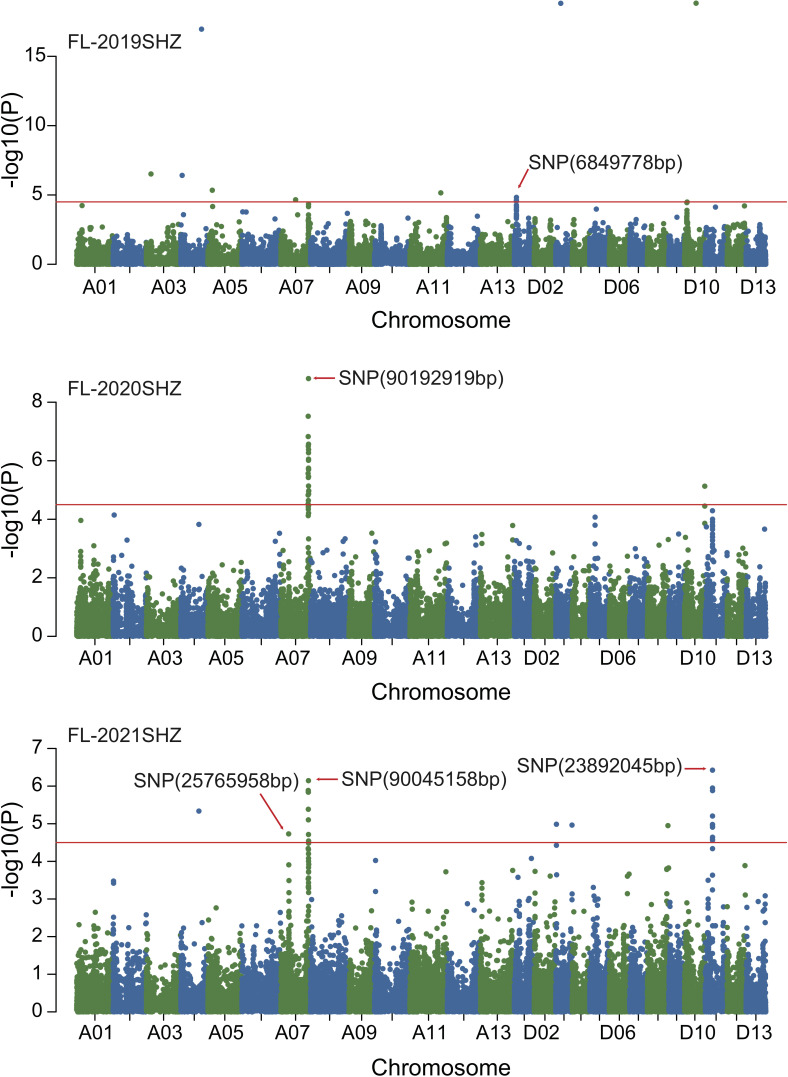
Manhattan of genome-wide association studies for fiber length in Shihezi. FL-2019SHZ: Fiber length in the Shihezi environment in 2019; FL-2020SHZ: Fiber length in the Shihezi environment in 2020; FL-2021SHZ: Fiber length in the Shihezi environment in 2021.

**Figure 3:**
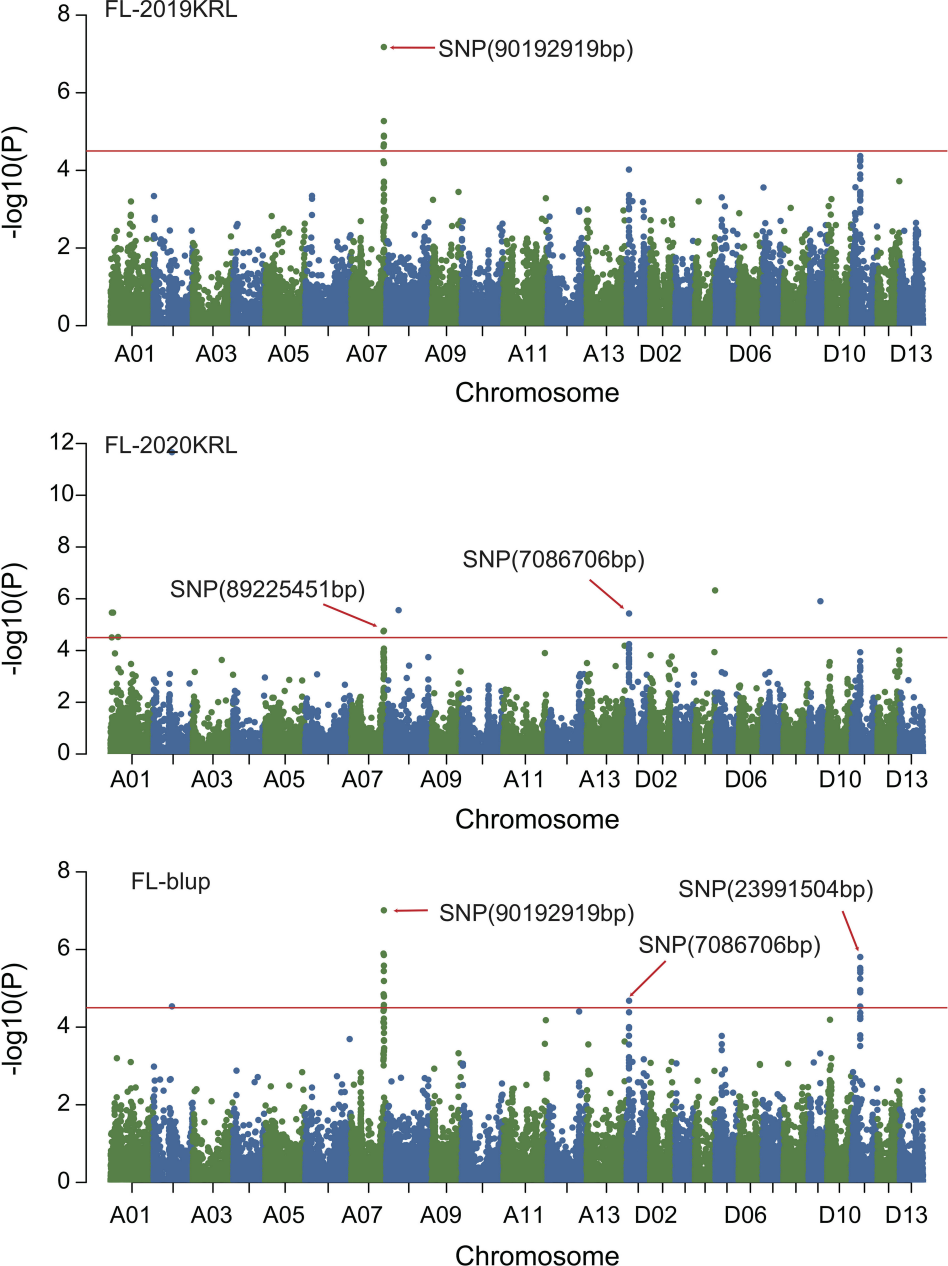
Manhattan of genome-wide association studies for fiber length in Korla. FL-2019KRL: Fiber length in the Korla environment in 2019; FL-2020KRL: Fiber length in the Korla environment in 2020; FL-blup: Fiber length in the blup environment.

**Figure 4:**
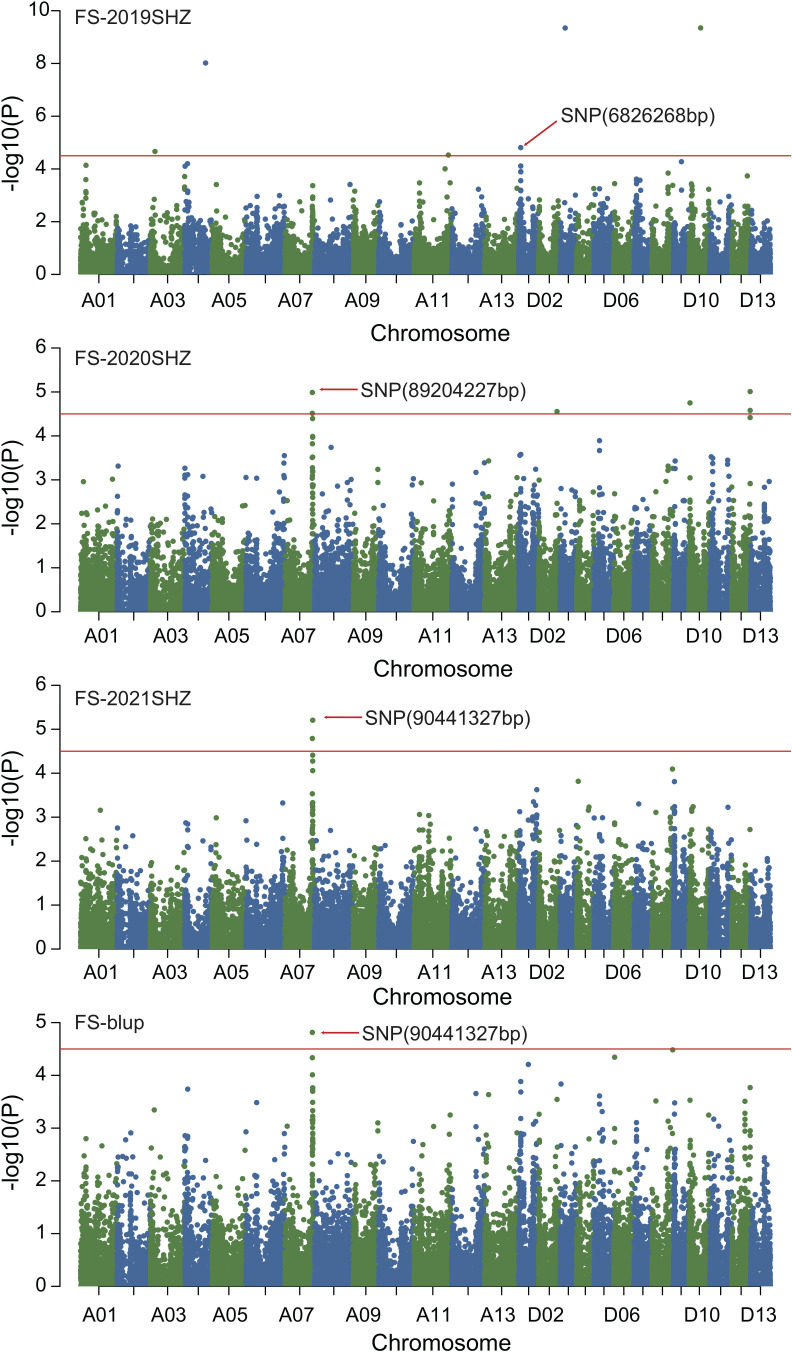
Manhattan of genome-wide association studies for fiber strength. FS-2019SHZ: Fiber strength in the Shihezi environment 2019; FS-2020SHZ: Fiber strength in the Shihezi environment 2020; FS-2021SHZ: Fiber strength in the Shihezi environment in 2021; FS-blup: Fiber strength in the blup environment.

**Figure 5:**
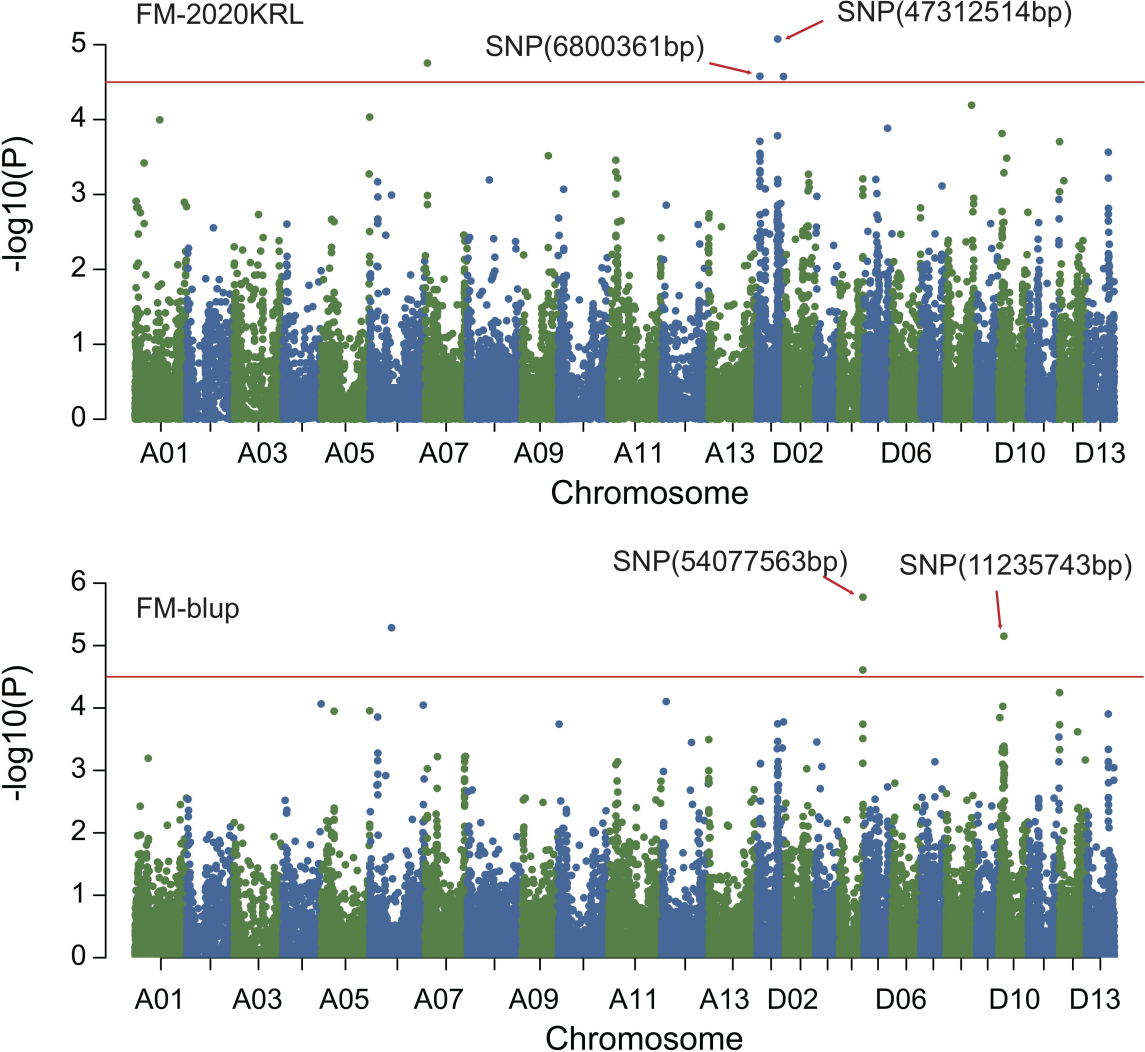
Manhattan of genome-wide association studies for micronaire. FM-2020KRL: Micronaire in the Korla environment in 2020; FM-blup: Micronaire in the blup environment in 2021.

Combined with the results of population analysis, GWAS were performed on the five fiber quality traits based on the mixed linear model method. A total of 42 SNP loci associated with the five fiber quality traits were detected beyond the significant threshold (−log10(*P*) ≥ 4.5) (**Table 3** and **Figures 2**, **3**, **4**, **5**). Among these 42 SNP loci, eight loci associated with fiber length were distributed on chromosomes A07, D01 and D11, five loci associated with fiber strength were distributed on chromosomes A07, A11 and D01, four loci related to micronaire were distributed on chromosomes D01, D04 and D10, and twelve loci associated with fiber uniformity were located on chromosomes A03, A05, A07, A08, A12, D09, D11, D12 and D13, whereas thirteen loci associated with fiber elongation were distributed on chromosomes A01, A03, A04, A05, A07, A09, D01, D03, D09 and D12. Seven loci were detected in more than two environments, including three loci related to fiber length which were distributed on chromosomes A07, D1 and D11, one locus related to fiber strength which was located on chromosome A07, two loci related to micronaire were distributed on chromosomes D4 and D10, and one locus related to fiber elongation was distributed on chromosome A03. However, on chromosome A07, the SNP locus (90192919bp) associated with the fiber length trait was detected in four environments, namely FL-2019KRL, FL-2020SHZ, FL-Blup and FL-Mean, whereas similar SNP loci (89225451bp and 90045158bp) were detected in the FL-2020KRL and FL-2021SHZ environments. The SNP (89225451bp) locus related to fiber strength was detected in the FS-blup and FS-mean environments, and adjacent SNP loci (892042771bp and 90441329bp) were detected in the FS-2020SHZ and FS-2021SHZ environments. On chromosome D01, the SNP (7086706bp) locus associated with the fiber length trait was detected in three environments, namely FL-2020KRL, FL-blup and FL-mean, and an adjacent SNP locus (6849778bp) was detected in the FL-2019SHZ environment, whereas a SNP locus (6826268bp) associated with fiber strength was detected only in the FS-2019SHZ environment. On chromosome D11, the SNP (23991504bp) locus associated with the fiber length trait was detected in the FL-blup and FL-mean environments, and same as SNP locus (23892045bp) which was detected in the FL-2021SHZ environment. All those signals related to fiber quality traits shed substantial light on the genomic basis of genetic improvement in cotton and will provide targets for molecular selection and genetic manipulation towards cotton improvement.

**Table 3:** Single-nucleotide polymorphic loci associated with fiber quality traits.

Trait	Chr.	Position (bp)	*P*-value	−log10 (*P*)	Environment
FL	A07	90192919	4.69E-08	7.33	FL_2019KRL
			8.48E-10	9.07	FL_2020SHZ
			7.60E-08	7.12	FL_blup
			6.85E-08	7.16	FL_mean
		89225451	3.12E-05	4.85	FL_2020KRL
		90045158	6.45E-07	6.20	FL_2021SHZ
		25765958	1.31E-05	4.88	FL_2021SHZ
	D01	7086706	2.85E-06	5.54	FL_2020KRL
			1.31E-05	4.88	FL_mean
			1.44E-05	4.84	FL_blup
		6849778	7.53E-07	6.12	FL_2019SHZ
	D11	23892045	4.14E-07	6.38	FL_2021SHZ
		23991504	1.56E-06	5.81	FL_blup
			1.85E-06	5.73	FL_mean
FS	A07	89204227	6.21E-06	5.21	FS_2020SHZ
		90441327	6.86E-06	5.16	FS_2021SHZ
		89225451	2.18E-05	4.66	FS_blup
			2.15E-05	4.67	FS_mean
	A11	113964819	7.45E-06	5.13	FS_2020KRL
	D01	6826268	1.36E-05	4.87	FS_2019SHZ
FM	D01	6800361	2.96E-05	4.63	FM_2020KRL
	D01	47312514	9.06E-06	5.04	FM_2020KRL
	D04	54077563	1.13E-06	5.95	FM_blup
			8.73E-07	6.06	FM_mean
	D10	11235743	1.34E-05	4.87	FM_blup
			1.36E-05	4.87	FM_mean
FU	A03	157029	1.31E-05	4.88	FU_2019SHZ
	A03	85682878	2.13E-05	4.67	FU_2019SHZ
	A05	14781291	1.87E-07	6.73	FU_2019SHZ
	A07	20771678	2.55E-05	4.59	FU_2020KRL
	A08	97346277	3.80E-07	6.42	FU_2019KRL
	A12	102656335	1.41E-05	4.85	FU_2019KRL
	D09	27075165	6.99E-08	7.16	FU_2019SHZ
	D09	22486814	1.01E-05	5.00	FU_2019KRL
	D11	9687979	1.78E-05	4.75	FU_2020SHZ
	D12	61385446	3.97E-08	7.40	FU_2019KRL
	D12	60465061	5.72E-06	5.24	FU_2020KRL
	D13	62662990	5.80E-06	5.23	FU_2019KRL
FE	A01	115726170	7.95E-06	5.10	FE_2019SHZ
	A03	157029	2.81E-06	5.55	FE_2019SHZ
	A03	853372	2.19E-05	4.66	FE_blup
			2.53E-05	4.60	FE_mean
	A04	7020433	3.07E-06	5.51	FE_2019KRL
	A05	14781291	6.00E-07	6.22	FE_2019SHZ
	A07	32497844	7.51E-06	5.12	FE_2019SHZ
	A09	60583506	2.34E-05	4.63	FE_2020KRL
	D01	6901867	1.27E-05	4.90	FE_2019SHZ
	D01	7029028	1.12E-05	4.95	FE_blup
	D03	16574571	5.78E-06	5.24	FE_mean
	D09	27075165	6.50E-08	7.19	FE_2019SHZ
	D12	53653807	2.42E-07	6.62	FE_2019SHZ
	D12	60046036	8.99E-07	6.05	FE_2020SHZ

## Discussion

### The use of “ZJU Cotton Array No. 1” 40K array and multi-environment trials improved the efficiency of genotyping and the accuracy of genome-wide association study results

The genotyping-by-target-sequencing (GBTS) technique was widely applied to maize, for which 20K and 40K chips were developed (Guo et al., 2019), and then developed for the rice 40K chip (Li X. W. et al.,2020) and the soybean “Zhongdouxin No.1” (ZDX1) chip (Liu et al., 2020). Liquid chips have been widely applied in population genetic diversity analyses (Du et al., 2019; Guo et al., 2021; Shaukat et al., 2021), genetic map construction, QTL mapping (Chen et al., 2019; Li et al., 2020) and gene mining (Gao et al., 2019). The “ZJU Cotton Array No. 1” 40K array developed by the GBTS technology and based on the cotton genome SNPs was used to genotype the targeted loci in the accessions in current research. Targeted genome sequencing greatly reduces the amount of DNA sequencing, obtains sufficient genotyping information and sufficient variations for GWAS, and simplifies bioinformatic analysis and data processing, thus improving detection efficiency and reducing the workload and cost of genotyping.

Accurate phenotypic data play a decisive role in GWAS results. The analysis of phenotypic variation for fiber quality traits in this study showed that the five traits exhibited differing degrees of variation in the five environments. Fiber strength was the trait most affected by the environment, whereas uniformity was the trait least affected by the environment. The five fiber quality traits interacted significantly with the environments. Multi-environment trials in the two different ecological regions, namely Korla (southern Xinjiang) and Shihezi (northern Xinjiang), improved the accuracy of phenotypic trait identification and reduced the interference of environmental factors on genetic evaluation, making the results of GWAS more accurate, reliable and precise.

### Population structure analysis improved the efficiency of parent selection in breeding

According to the analysis of the genotyping array results, 612 upland cotton accessions from the Yellow River Basin cotton-producing region, the Yangtze River Basin cotton-producing region, the northern extra-early-maturing region, the Northwest inland cotton-producing region and accessions from abroad were divided into four groups. Nearly 60% of the accessions from the Yellow River Basin, the Yangtze River Basin and from abroad were clustered in the second group, whereas the accessions from the Northwest inland cotton region were distributed in all four groups, but mainly in the third group, where they were relatively concentrated. Genetic relationship analysis showed that the cultivars in China showed highly narrow kinship and the accessions from the Northwest inland cotton-producing region were distributed in various groups, and had close genetic relationship with the accessions from other cotton-producing regions in China, which were highly similar with previous researches (Tian, 2016; Han et al., 2020; Han et al., 2022). Therefore, in cotton breeding practice, elite cotton parents should be selected from different groups to expand the source of variation to integrate elite alleles to construct a strong genetic background; on the other hand, the mining and identification of elite genetic resources related to fiber quality should be increased in order to develop new varieties adapted to cotton-growing conditions in China and capable of producing high yields of high-quality fiber.

### Stable and aggregated SNP loci have important applications to molecular breeding of cotton

In the current study, a total of 42 SNP loci related to fiber length, fiber strength, micronaire, uniformity and elongation were detected, seven of which were detected in more than two environments. These 42 SNP loci were distributed on 17 chromosomes, nine of which were on subgroup A chromosomes, namely A01, A03, A04, A05, A07, A08, A09, A11 and A12, and eight of which were on subgroup D chromosomes, namely D01, D03, D04, D09, D10, D11, D12, and D13.

Compared with previous study (Han et al., 2022), two loci related to fiber elongation, D12:53653807and D12:60046036, were located in adjacent location (D12:53455112 and D12:61156498); For fiber strength, there were four loci (A07:89204227, A07:89225451, A07:90441327, A11:113964819) near to the signals A07:89012271, A07:90524406 and A11:113990127; and for fiber uniformity, one locus A12:102656335 was nearby the site A12:100857997 and A12:105646774, suggesting the highly consistent with the whole genome resequencing research. Moreover, five SNP loci were detected on chromosome D11, including one locus related to fiber uniformity, two loci related to micronaire, and two loci related to fiber elongation, were closely consistent with multiple researches on different populations that there was a hotspot signal associated with fiber quality on chromosome D11 (Wang et al., 2017; Ma et al., 2018; Li, Z. H. et al., 2020; Han et al., 2022).

Therefore, 21 of the 42 SNP loci were distributed on chromosomes A07, D01 and D11. These three chromosomes had the highest correlation with fiber quality traits and the aggregation of loci, to which attention should be paid with respect to breeding for high-quality fiber. On chromosome A07, the SNP locus (90192919bp) associated with fiber length was stable, being detected in four environments, and adjacent loci (90045158bp, 89225451bp) were also detected in two environments, whereas adjacent loci (89204227bp, 90441327bp, 89225451bp) associated with fiber strength were detected in three environments, indicating that these trait-related signals were relatively stable expressed in different environments, and could respond to focused selection in breeding for high fiber quality in cotton.

## Conclusion

Precise genotype and phenotype identification are prerequisite for improving the accuracy of genome-wide association study results. A total of 612 upland cotton accessions were genotyped using the “ZJU Cotton Chip No. 1 40K” chip array *via* the liquid-phase probe hybridization-based genotyping-by-target-sequencing technology. Population structure analysis divided the 612 accessions into four groups illuminated the narrow kinship in Chinese cultivars and the close relationship between the accessions from Northwest inland cotton-producing region and other cotton-planting regions in China. Eventually, GWAS analysis of five fiber quality traits in five environments was performed and a total of 42 SNP loci associated with fiber quality was detected. Our results could provide more precise molecular markers and genetic resources for cotton breeding for better fiber quality in the future.

## Data availability statement

The datasets presented in this study can be found in online repositories. The names of the repository/repositories and accession number(s) can be found at the National Center for Biotechnology Indormation under the accession PRJNA744011 and the cotton database website http://cotton.zju.edu.cn.

## Author contributions

QS and ZS designed and supervised the research. HC, ZH, QM, CD, XN, JL, HL, ZS, and YL conducted the field trials to evaluate the traits. HC, ZH, ZS, and YL performed the data analysis. HC and ZH wrote the manuscript. All of the authors read and approved the manuscript.

## Funding

This research was funded by The Science Technology and Achievement Transformation Project of The Xinjian Production and Construction Corps (2019AB021, 2021AB008) and Innovating Team (2020CB003).

## Conflict of interest

The authors declare that the research was conducted in the absence of any commercial or financial relationships that could be construed as a potential conflict of interest.

## Publisher’s note

All claims expressed in this article are solely those of the authors and do not necessarily represent those of their affiliated organizations, or those of the publisher, the editors and the reviewers. Any product that may be evaluated in this article, or claim that may be made by its manufacturer, is not guaranteed or endorsed by the publisher.
