# Supplementary material for: Identification of elite fiber quality loci in upland cotton based on the genotyping-by-target-sequencing technology
Source: Front Plant Sci. 2022 Nov 3;13:1027806. doi: 10.3389/fpls.2022.1027806 (PMC9669494; doi:10.3389/fpls.2022.1027806)
Supplement: Supplementary file 1 [file DataSheet_1.docx]

**Supplementary material**

**Supplementary Table 1. Statistical analysis for the variability of fiber quality traits**

| **Trait** | **Environment** | **Min** | **Max** | **Mean** | **SD** | **CV (%)** |
| --- | --- | --- | --- | --- | --- | --- |
| FL | 2019SHZ | 21.34 | 36.45 | 29.42 | 1.83 | 6.22 |
|  | 2019KRL | 19.10 | 34.43 | 27.11 | 2.39 | 8.82 |
|  | 2020SHZ | 20.97 | 35.17 | 28.60 | 1.91 | 6.68 |
|  | 2020KRL | 21.44 | 35.93 | 28.98 | 1.98 | 6.83 |
|  | 2021SHZ | 25.61 | 36.70 | 30.83 | 1.80 | 5.84 |
|  | Mean | 21.70 | 34.74 | 28.99 | 1.98 | 6.83 |
| FS | 2019SHZ | 19.60 | 48.50 | 32.19 | 3.38 | 10.50 |
|  | 2019KRL | 21.20 | 42.50 | 28.10 | 3.23 | 11.49 |
|  | 2020SHZ | 20.19 | 40.57 | 30.34 | 3.22 | 10.61 |
|  | 2020KRL | 20.78 | 40.87 | 29.56 | 3.16 | 10.69 |
|  | 2021SHZ | 23.13 | 45.28 | 33.37 | 3.53 | 10.58 |
|  | Mean | 20.98 | 43.54 | 30.71 | 3.30 | 10.75 |
| FM | 2019SHZ | 2.70 | 5.67 | 4.37 | 0.40 | 9.15 |
|  | 2019KRL | 2.65 | 5.54 | 3.92 | 0.38 | 9.69 |
|  | 2020SHZ | 3.00 | 5.73 | 4.31 | 0.36 | 8.35 |
|  | 2020KRL | 2.85 | 5.78 | 4.29 | 0.37 | 8.62 |
|  | 2021SHZ | 2.64 | 5.92 | 4.52 | 0.51 | 11.28 |
|  | Mean | 2.77 | 5.73 | 4.28 | 0.40 | 9.42 |
| FU | 2019SHZ | 77.40 | 89.20 | 85.20 | 1.02 | 1.20 |
|  | KRL2019 | 75.20 | 89.50 | 84.17 | 1.50 | 1.78 |
|  | 2020SHZ | 77.30 | 89.10 | 84.34 | 1.39 | 1.65 |
|  | 2020KRL | 76.60 | 88.70 | 84.18 | 1.48 | 1.76 |
|  | 2021SHZ | 79.00 | 88.90 | 85.87 | 1.17 | 1.36 |
|  | Mean | 77.1 | 89.08 | 84.75 | 1.31 | 1.55 |
| FE | 2019SHZ | 6.30 | 7.30 | 6.79 | 0.11 | 1.62 |
|  | 2019KRL | 5.20 | 7.20 | 6.72 | 0.20 | 2.98 |
|  | 2020SHZ | 6.10 | 7.00 | 6.62 | 0.11 | 1.66 |
|  | 2020KRL | 6.10 | 7.10 | 6.69 | 0.12 | 1.79 |
|  | 2021SHZ | 6.30 | 7.40 | 6.83 | 0.14 | 2.05 |
|  | Mean | 6.00 | 7.20 | 6.73 | 0.14 | 2.08 |

2019SHZ:the Shihezi environment in 2019; 2019KRL: the Korla environment in 2019; 2020SHZ:the Shihezi environment in 2020; 2020KRL: the Korla environment in 2020; 2021SHZ:the Shihezi environment in 2021.

**Supplementary Table 2. Statistical analysis for the variance and the heritability for fiber quality traits**

| **Trait** | **Source** | **Sum of square** | **Degree of freedom** | **Mean square** | **Variance** | **Significance** | **Heritability（%）** |
| --- | --- | --- | --- | --- | --- | --- | --- |
| FL | Accession | 41165.81 | 611 | 67.37 | 28.21 | ** | 84.94 |
|  | Environment | 6821.21 | 4 | 1705.30 | 713.92 | ** |  |
|  | Accession × environment interaction | 20568.88 | 2444 | 8.42 | 3.52 | ** |  |
|  | Error | 14618.58 | 6120 | 2.39 | - |  |  |
| FS | Accession | 85540.70 | 611 | 140.00 | 34.66 | ** | 87.07 |
|  | Environment | 17776.82 | 4 | 4444.20 | 1100.25 | ** |  |
|  | Accession × environment interaction | 31655.82 | 2444 | 12.95 | 3.21 | ** |  |
|  | Error | 24720.22 | 6120 | 4.04 | - |  |  |
| FM | Accession | 951.76 | 611 | 1.56 | 21.12 | ** | 95.25 |
|  | Environment | 280.34 | 4 | 70.09 | 950.14 | ** |  |
|  | Accession × environment interaction | 448.48 | 2444 | 0.18 | 2.49 | ** |  |
|  | Error | 451.43 | 6120 | 0.07 | - |  |  |
| FU | Accession | 22976.42 | 611 | 37.61 | 5.11 | ** | 45.10 |
|  | Environment | 2232.84 | 4 | 558.21 | 75.84 | ** |  |
|  | Accession × environment interaction | 39330.65 | 2444 | 16.09 | 2.19 | ** |  |
|  | Error | 45043.78 | 6120 | 7.36 | - |  |  |
| FE | Accession | 199.74 | 611 | 0.33 | 6.40 | ** | 50.49 |
|  | Environment | 43.41 | 4 | 10.85 | 212.40 | ** |  |
|  | Accession × environment interaction | 260.39 | 2444× | 0.11 | 2.09 | ** |  |
|  | Error | 312.73 | 6120 | 0.05 | - |  |  |

* and ** indicate significance at *P*=0.05 and *P*=0.01,levels, respectively.


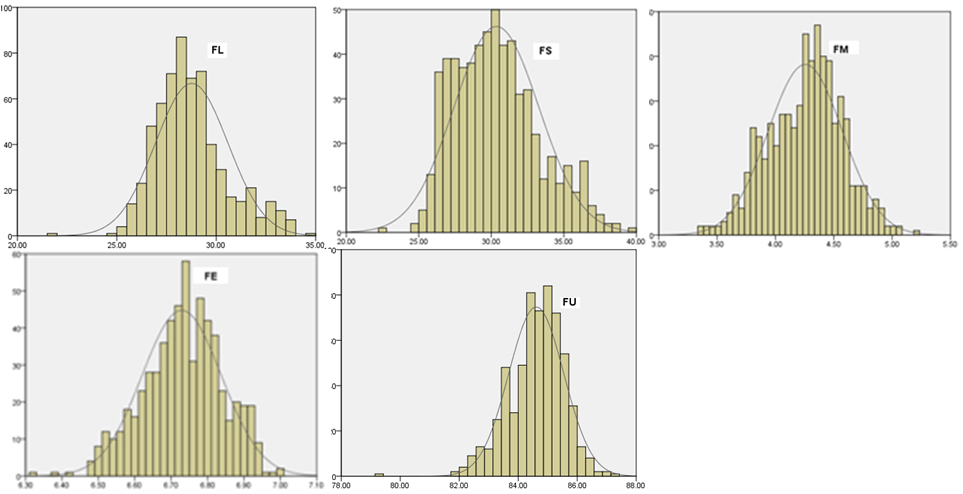


**SUPPLEMATARY FIGURE 1. Normal distribution of fiber quality traits**
